# Topography of cranial foramina and anaesthesia techniques of cranial nerves in selected species of primates (Cebidae, Cercopithecidae, Lemuridae) – part I – osteology

**DOI:** 10.1186/s12917-023-03680-7

**Published:** 2023-08-12

**Authors:** Jan Bold, Michalina Szemet, Karolina Goździewska-Harłajczuk, Maciej Janeczek, Joanna E. Klećkowska-Nawrot

**Affiliations:** Department of Biostructure and Animal Physiology, Wrocław University of Environmental and Life Sciences, Wrocław, Poland 51-630, Kozuchowska 1,

**Keywords:** Primates, Cranial nerves, Block anaesthesia

## Abstract

**Background:**

Conductive anaesthesia of the nerves around the head is one of the methods of intraoperative pain relief (under deep anaesthesia but before proceeding with the procedure). Performing this procedure on primates is especially challenging for the veterinarian, due to their cranial anatomy and topography, which has more in common with the human skull than with the skulls of other animals. Knowledge of key bony structures, including cranial foramina, is essential for effective anaesthesia of the cranial nerves.

**Results:**

In this study, the differences in the topography of the cranial foramina in eight selected species of primates were examined: Angola colobus (*Colobus angolensis*), Celebes crested macaque (*Macaca nigra*), L’Hoest’s monkey (*Allochrocebus lhoesti*), baboon (*Papio cynocephalus*), buff-bellied capuchin (*Sapajus xanthosternos*), black-and-white ruffed lemur (*Varecia variegata*), crowned lemur (*Eulemur coronatus*), and a ring-tailed lemur (*Lemur catta*) coming from the Wroclaw Zoological Garden (Poland). The cranial nerves running through the foramina have also been described and their anaesthesia techniques against bone points have been tested to relieve post-operative pain in the area of the head supplied by these nerves.

**Conclusion:**

The tests carried out show differences in the topography of the cranial foramina, and therefore also differences in the methods of injection, so the results obtained in this study may be useful in veterinary medicine, especially for practising veterinarians.

## Background

Cranial nerves that originate in the brain pass through openings in the skull and they innervate various tissues and organs of the head and its surroundings. The foramina differ in their structure, topography and size depending on the animal species and individual characteristics and they are a reference point for the veterinarian to perform perineural injections, for example, to relieve pain while reducing the dose of systemic anaesthetic. This reduces the risk of complications and the patient’s recovery time [1–3]. Regional anaesthesia is one of the methods of local anaesthesia and it consists in blocking peripheral nerve conduction. These methods are used in veterinary dentistry in many species of domestic animals as well as in exotic animals. All procedures are performed with great success, e.g. in dogs and cats [3, 4]. The possibility of performing head anaesthesia in other animals has also been described, for example, dromedary camel (*Camelus dromedarius*) [5], Eurasian wild boar (*Sus scrofa*) [6], domestic cattle (*Bos taurus*) [5], New Zealand rabbit [7]. In addition, studies on the injections in primates can be found in the available literature, for example in the king macaque (*Macaca mulatta*) [8]. This method creates optimal operating conditions and ensures long-term postoperative analgesia, even at the stage of performing the procedure [9]. When it is a part of mixed anaesthesia (including general anaesthesia) is referred to as multimodal analgesia [10, 11]. It consists in applying a local anaesthetic in the vicinity of the nerve trunk. Based on this knowledge, is possible to obtain tissue anaesthesia in the entire area innervated by a given nerve with one injection, using a small amount of anaesthetic. In addition, when local anaesthesia is used, the need for systemic drugs decreases, which is tantamount to an increase in the safety of anaesthesia [12]. Another aspect used by doctors when using this method of anaesthesia is the reduced cortical facilitation of pain stimuli, which has a positive effect on the intra- and postoperative need for analgesics [1, 10]. According to the literature on human dentistry, local anaesthetics are the safest and most effective drugs for the prevention and treatment of pain in dental treatment, provided they are used correctly [13]. For the same reason, veterinarians are increasingly turning to local anaesthesia techniques as a supplement to general anaesthesia in their patients [10, 14–18].

The basic problem with conduction anaesthesia (nerve blocks) is the fact that the anaesthetic should not be administered into the nerve trunk itself - this can lead to serious complications, including permanent nerve damage. In addition, perineural injections carry the risk of hematoma, peripheral neuritis, or abscess [19]. Depending on the nerve being anaesthetized, complications may include dysphonia, laryngospasm, paralysis of the vocal cords, and feeling unable to breathe [13, 20]. The drug should be deposited near the nerve [10, 12] and for this reason, the knowledge of the topographic anatomy of a given area by the surgeons who are to perform given anaesthesia is so important. Due to the closer anatomical similarity of the studied species of primates to humans than to other mammals, the techniques of anaesthesia of these cranial nerves, adapted from the human medical literature, are presented below.

The research aimed to learn the topography of the skull of primates, paying attention to the foramina from which the cranial nerves exit. Knowledge of bone structures is necessary for the effective use of anaesthesia because some injections used in other animal species are not applicable in primates precisely because of the characteristic structure of the skull. The skull publication is only the first in a series. Part II will concern the use of anaesthesia with attention to the anatomical structures of soft tissues, i.e. major salivary glands, lymph nodes, blood vessels and nerves. Knowledge about the anatomy of above mentioned structures and the described procedures will allow veterinarians to apply the appropriate method of anaesthesia block to the appropriate nerve. Such data on exotic primates, while not necessarily of use to a general practitioner, is especially important for veterinarians practising in zoos, primate sanctuaries and national parks, and with primate researchers. However because a limited number of individuals were available in some instances the proposed routes for and placement of needles should be interpreted as guidance and less as a directive.

## Materials and methods

**Fig. 1:**
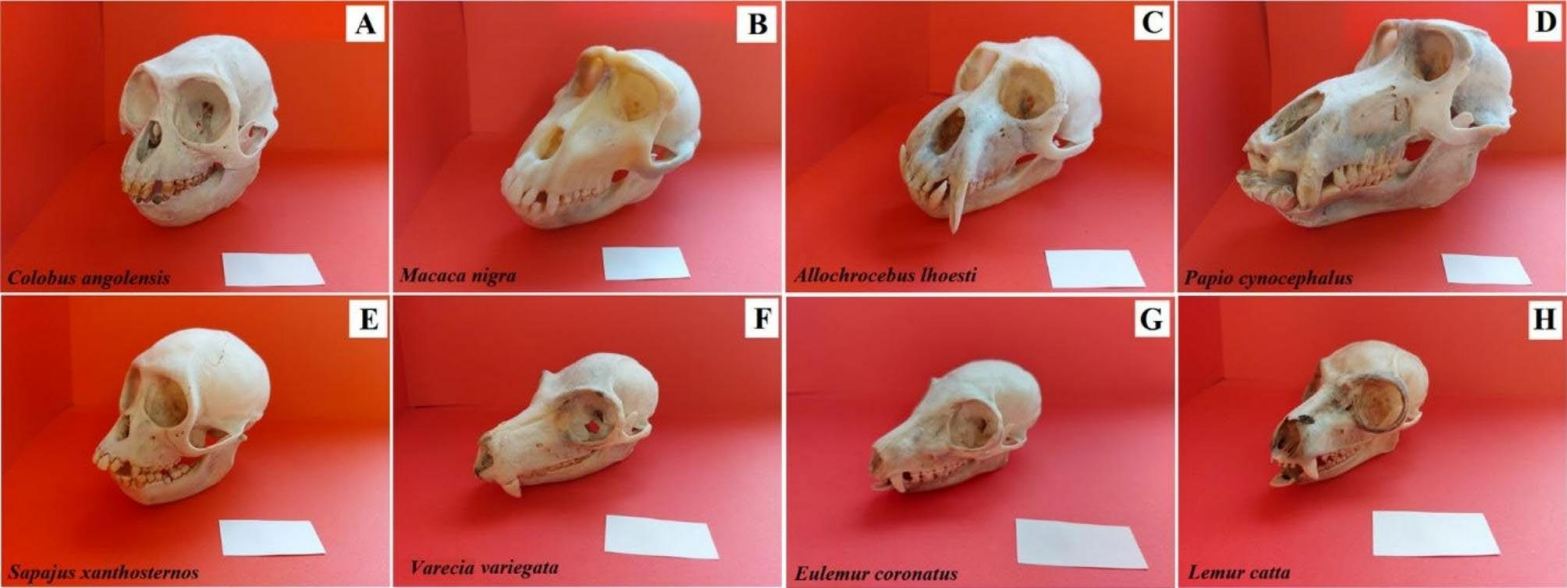
Photograph of the skull of examined Primates. **A** – Angola colobus (*Colobus angolensis*), **B** – Celebes crested macaque (*Macaca nigra*), b – L’Hoest’s monkey (*Allochrocebus lhoesti*), **D** – Baboon (*Papio cynocephalus*), **E** – Buff-bellied capuchin (*Sapajus xanthostrenos*), **F** – Black-and-white ruffed lemur (*Varecia variegata*), **G** – Crowned lemur (*Eulemur coronatus*), **H** – Ring-tailed lemur (*Lemur catta*). Bar = 2 cm

**Collection of Specimen and Conservation status.** The study material compared eight adult captive species of primates: yellow Angola colobus (*Colobus angolensis*), Celebes crested macaque (*Macaca nigra*), L’Hoest’s monkey (*Allochrocebus lhoesti*), baboon (*Papio cynocephalus*), buff-bellied capuchin (*Sapajus xanthosternos*), black-and-white ruffed lemur (*Varecia variegata*), crowned lemur (*Eulemur coronatus*), and a ring-tailed lemur (*Lemur catta*) coming from the Wroclaw Zoological Garden (Poland). These animals were collected in the years 2016–2022 in the Division of Animal Anatomy of the Department of Biostructure and Animal Physiology, Faculty of Veterinary Medicine, Wroclaw University of Environmental and Life Sciences (Fig. 1). All animals were not euthanized for current study (natural death of animals) and were obtained post-mortem. Systematics, status to the IUCN Red List of Threatened Species (2022) and the number of specimens tested are presented in Table 1.

**Table 1:** Primates described in present study and their conservation status according to the IUCN Red List of Threatened Species (2022) [21]

ORDER	SUBORDER	INFRAORDER	FAMILY	GENUS	SPECIES	CONSERVATION STATUS	NUMBER OF ANIMALS
Primates	Haplorhini	Simiiformes	Cercopithecidae	*Colobus*	Angola colobus (*Colobus angolensis* Sclater, 1860)	VU Decreasing	1
				*Macaca*	Celebes crested macaque (*Macaca nigra* Desmarest, 1822)	CR Decreasing	1
				*Allochrocebus*	L’Hoest’s monkey (*Allochrocebus lhoesti* Sclater, 1899)	VU Decreasing	2
				*Papio*	baboon (*Papio cynocephalus* Linnaeus, 1766)	LC Stable	7
			Cebidae	*Sapajus*	buff-bellied capuchin (*Sapajus xanthosternos* Wied, 1826)	CR Decreasing	1
	Strepsirrhini	–	Lemuridae	*Variecia*	black-and-white ruffed lemur (*Varecia variegata* Kerr, 1792)	CR Decreasing	2
				*Elemur*	crowned lemur (*Eulemur coronatus* Gray, 1842)	EN Decreasing	1
				*Lemur*	ring-tailed lemur (*Lemur catta* Linnaeus, 1758)	EN Decreasing	8

**Fig. 2:**
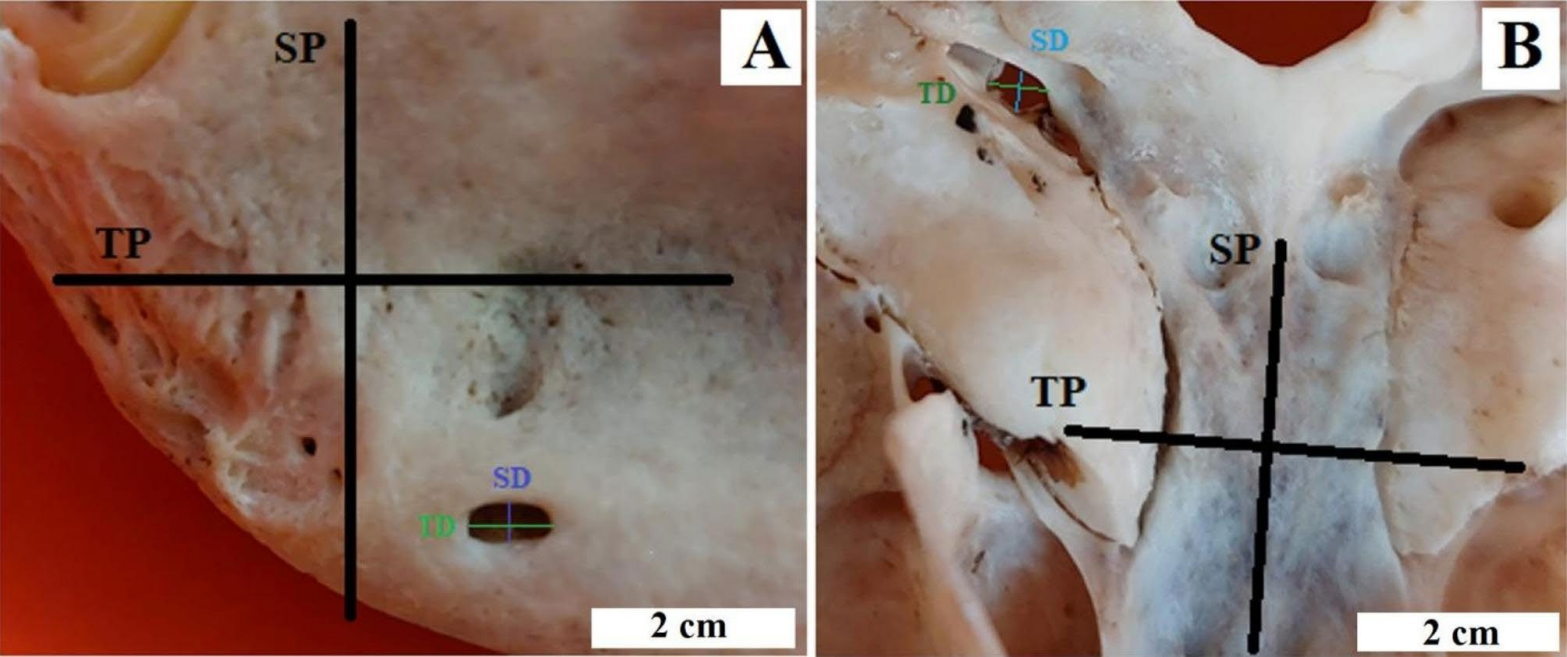
Method of the cranial foramina measurement. SP (sagittal plane) – sagittal plane, TP (transverse plane) – horizontal plane, SD (sagittal diameter) - length, TD (transverse diameter) - width. **A** - Celebes crested macaque (*Macaca nigra*) (*foramen mentale*); **B** - L’Hoest’s monkey (*Allochrocebus lhoesti*) (*foramen jugulare*)

**Procedures.** The collected animal bodies were dissected, the skin was incised with a scalpel in the neck area and the muscles, trachea, oesophagus and large blood vessels at the level of the atlanto-occipital joint were cut. A scalpel blade was inserted between the articular surfaces of the atlas and occipital condyles, then the bones were separated and the head was dissected from the trunk. The skin and most of the soft tissues were removed from the head and the skulls were boiled with detergent (fabric washing powder). The thermal dissection was completed when the remains of soft tissues were imperceptible on the bones. The skulls were then cleaned in clean running water and left in a ventilated room to dry completely [22]. After a few days, the macroscopic evaluation of the skulls began, and then they were photographed. After examining the cranial foramina, their topographies about other bone structures present on the skull were described, castings of the foramina (for easier repeatable measurements) were made using a thermoplastic modelling material and the dimensions of these structures were measured. Two different measurements of each cast were made and the values were vertical (length - in line with the sagittal plane of the skull) and horizontal (width - in line with the horizontal plane of the skull) (Fig. 2). A digital calliper (Stainless Hardened, Farnell, Poland) was used to measure with an accuracy of 0.01 mm [23]. Values were obtained from both sides of the skull (right and left). Each length and width were measured twice to reduce the risk of statistical error. Due to the presence of many foramina of the same type on one skull, all the same values from the foramina belonging to the same group were summed up and then divided by the number of foramina (the corresponding values of foramina from a given species were summed up and divided by the number of individuals from a given species). The results obtained are presented in Table 2. After the metric examination, the injection methods were adapted according to the described anatomy, the injection technique was described and the cervical openings without the needle and other examined openings were photographed, taking into account the inserted needle according to the described anaesthesia technique.

**Table 2:** Morphometry of the cranial foramina in eight primates species (values are expressed in mm)

Species	foramen	*f. jugulare*		*f. infraorbitale*		*fissura* *pterygopalatina*		*f. ovale*		*f. mentale*		*f. mandibulae*		*f. palatinum* *majus*	
	value	right	left	right	left	right	left	right	left	right	left	right	left	right	left
**Black-and-white ruffed lemur (** ***Varecia variegata*** **)**	length	1.66	1.40	1.03	1.28	absence		2.92	2.66	0.84	0.73	1.98	1.64	1.44	1.78
	width	1.38	1.34	1.03	1.33			2.26	1.92	0.67	0.75	1.68	1.54	1.24	1.38
**Buff-bellied capuchin (** ***Sapajus xanthosternos*** **)**	length	1.89	1.97	0.94	1.21	8.77	8.33	3.27	3.43	0.85	0.83	1.72	1.74	0.89	0.85
	width	1.36	1.44	0.87	1.11	4.71	4.94	1.27	1.76	0.80	0.76	1.13	1.08	0.73	0.65
**Baboon (** ***Papio cynocephalus*** **)**	length	3.08	3.28	1.51	1.43	14.44	14.28	4.38	3.48	1.36	1.56	6.20	6.64	5.54	5.22
	width	5.12	5.24	1.09	1.38	5.08	5.94	6.50	6.88	1.64	1.72	3.34	2.36	3.28	3.54
**Angola colobus (** ***Colobus angolensis*** **)**	length	2.24	1.76	1.54	1.26	8.98	8.88	5.62	5.64	1.36	0.87	2.42	2.36	1.28	3.16
	width	2.40	2.50	1.72	1.69	3.26	2.54	2.22	2.38	1.06	0.85	1.48	1.38	1.04	1.12
** L’Hoest’s monkey (** ***Allochrocebus lhoesti*** **)**	length	1.74	2.02	1.46	1.40	8.22	8.96	3.24	3.56	1.04	1.18	3.04	3.30	3.38	3.26
	width	1.80	1.76	1.83	1.45	3.44	2.66	1.08	1.18	1.62	2.20	2.08	1.78	1.44	1.40
**Celebes crested macaque (** ***Macaca nigra*** **)**	length	2.84	3.00	0.86	1.20	12.06	11.56	2.46	2.04	1.15	1.06	4.08	3.84	2.46	2.46
	width	2.58	2.80	0.92	0.93	3.40	2.68	5.86	5.26	0.91	0.98	2.56	3.10	1.54	1.88
**Ring-tailed lemur (** ***Lemur catta*** **)**	length	2.04	2.22	2.22	1.78	absence		2.16	2.46	0.74	0.85	2.10	2.56	1.70	2.12
	width	1.54	1.78	1.16	1.22			1.76	2.00	0.61	0.60	1.58	0.98	1.28	1.48
**Crowned lemur (** ***Eulemur coronatus*** **)**	length	2.56	2.66	1.98	1.94	absence		2.04	2.16	0.97	1.03	1.68	1.64	1.10	1.56
	width	2.06	2.06	0.78	0.90			1.90	2.24	0.87	1.03	1.38	1.18	0.60	0.78

## Results and discussion

**Topography of the cranial foramina.**

**Fig. 3:**
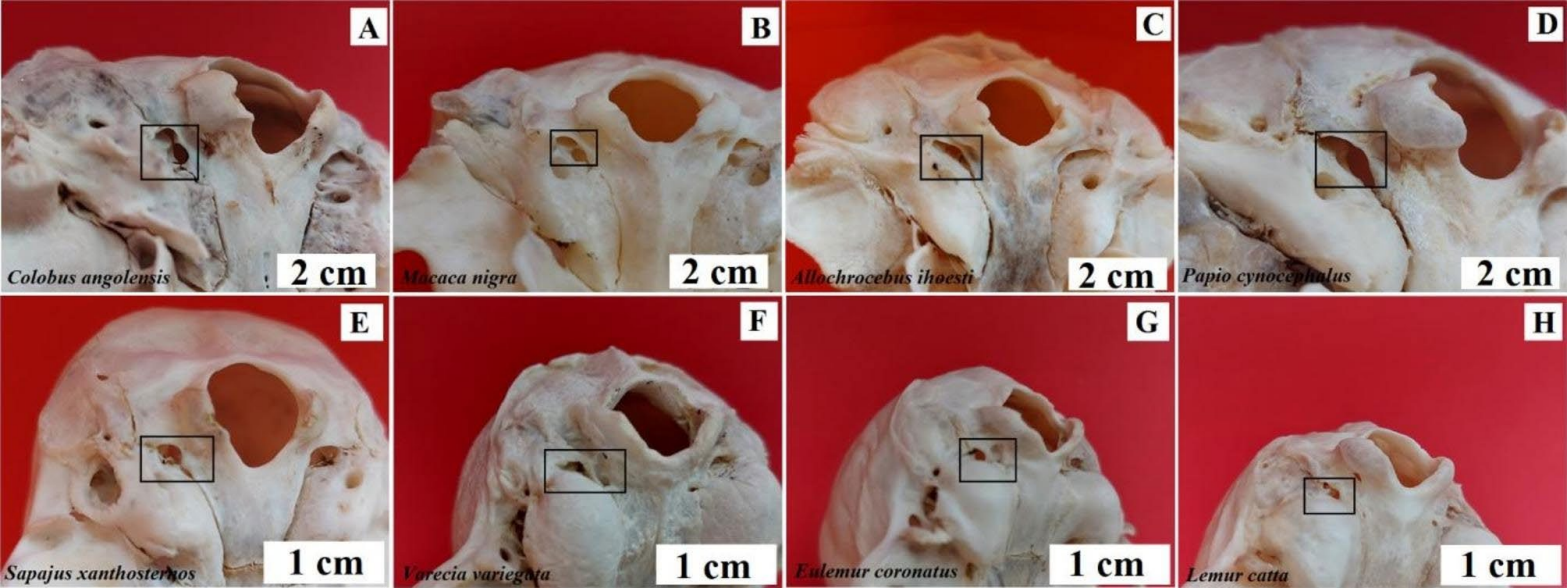
Topography of the foramen jugulare. **A** – Angola colobus (*Colobus angolensis*), **B** – Celebes crested macaque (*Macaca nigra*), **C** – L’Hoest’s monkey (*Allochrocebus lhoesti*), **D** – Baboon (*Papio cynocephalus*), **E** – Buff-bellied capuchin (*Sapajus xanthostrenos*), **F** – Black-and-white ruffed lemur (*Varecia variegata*), **G** – Crowned lemur (*Eulemur coronatus*), **H** – Ring-tailed lemur (*Lemur catta*)

The jugular foramen (*foramen jugulare*) is located on the border of the basilar part of the occipital bone and the temporal bone on both sides of the foramen magnum. In the centre of the jugular opening is the intracervical process (*processus intrajugularis*), which divides it into two parts. The medial part - relatively smaller - contains the glossopharyngeal nerve (*nervus glossopharyngeus*), the lateral part – relatively larger – contains the vagus nerve (*nervus vagus*) and the accessory nerve (*nervus accessorius*) [24–27]. In baboons, buff-bellied capuchins, L’Hoest’s monkey, Angola colobus and Celebes crested macaque, the orifice of the foramen is directed basolateral-ventrally (Fig. 3). In the black-and-white ruffed lemur, ring-tailed lemur, and crowned lemur, the intracervical process protrudes above the foramen space, clearly dividing into a medial caudal and lateral ventral portion (Fig. 3).

The infraorbital foramen (*foramen infraorbitale*) is located on the anterior surface of the maxillary body below the infraorbital margin and there may be more than one foramen. From it comes the infraorbital nerve (*nervus infraorbitalis*) - a branch of the maxillary nerve. The infraorbital nerve gives off the superior alveolar branches responsible for the innervation of the maxillary teeth and the terminal branches innervating the skin and mucosa of the nose, upper lip and lower eyelid [24, 28, 29]. In all monkeys, the orifice of the foramen is directed nasally, the baboon has six foramina on the right side and three foramina on the left side, located at the level of the fourth and fifth cheek teeth, Celebes crested macaque has five foramina on each side at the level of the fourth cheek tooth. The Angola colobus has three foramina on each side, and the black-and-white ruffed lemur has four foramina on the right and two on the left - in both monkeys, they are located at the level of the third and fourth cheek teeth. L’Hoest’s monkey has three foramina on each side at the level of the fourth cheek tooth. At the same height, the infraorbital foramen of the ring-tailed lemur is located, one on each side of the jaw. The presence of one opening on both sides was observed in crowned lemurs at the height of the second cheek tooth. In the buff-bellied capuchin, there were also two foramina on each side at the level of the second cheek tooth.

The greater palatine foramen (*foramen palatinum major*) is present in the posterior part of the palatal surface of the horizontal plate of the palatine bone, and the greater palatine nerve (*nervus palatinus major*) emerges from it [24, 30, 31]. In all monkeys, this opening was directed rostrally and in the baboon, black-and-white ruffed lemur, buff-bellied capuchin, ring-tailed lemur, crowned lemur, and Celebes crested macaque, it was just past the last cheek tooth. In L’Hoest’s monkey and Angola colobus was located at the level of this tooth.

The mandibular foramen (*foramen mandibulae*) is located on the medial side of the mandibular branch and is the beginning of the mandibular canal. The inferior alveolar nerve (*nervus alveolaris inferior*) runs through it, originating from the mandibular nerve (*nervus mandibularis*), which passes into the mental nerve (*nervus mentalis*) and exits through the mental foramen (*foramen mental*). There may be more than one mental foramen and more than one mental nerve [24, 28, 32]. Baboons had five foramina on each side of the mandible, Celebes crested macaque and buff-bellied capuchin had two foramina, and ring-tailed lemur and crowned lemur had three foramina on each side of the mandible, Angola colobus showed two foramina on the left and one on the right, the black-and-white ruffed lemur had five foramina on the left and four foramina on the right, and L’Hoest’s monkey had one foramen on the left and two foramina on the right. The mandibular foramen of a baboon, buff-bellied capuchin, L’Hoest’s monkey, Angola colobus, and Celebes crested macaque was located at the level of the rostral end of the coronoid process of the mandible with the orifice directed caudodorsally. In the black-and-white ruffed lemur, ring-tailed lemur, and crowned lemur, this opening is present at the level of the rostral end of the coronoid process of the mandible, and the opening is directed caudally.

The pterygopalatine fossa (*fossa pterygopalatina*) is located between the jawbone and the sphenoid bone. It connects with other cranial spaces through numerous openings and canals: sphenoid-palatine foramen, inferior orbital fissure, circular foramen, pterygoid canal, palato-vaginal canal, and greater palatal canal. The pterygopalatine fossa is bounded: superiorly by the body of the sphenoid bone, anteriorly by the body of the maxilla and the orbital process of the palatine bone, posteriorly by the pterygoid process of the sphenoid bone and the greater wing of the sphenoid bone, laterally by the pterygopalatine fissure (*fissura pterygopalatina*), medially by the vertical plate of the pterygopalatine bone. It passes from below into the greater palatal canal. The most important nerve structures present in the pterygopalatine fossa are the pterygopalatine ganglion (*ganglion pterygopalatinum*) and the maxillary nerve (*nervus maxillaris*) [24, 25, 33]. A pterygopalatine fissure is absent in the black-and-white ruffed lemur, ring-tailed lemur, and crowned lemur.

The oval foramen (*foramen ovale*) is located on both sides of the skull in the greater wing of the sphenoid. The mandibular nerve (*nervus mandibularis*) emerges from the oval foramen and runs nasally-inferiorly giving branches to the masticatory nerve (*nervus masticatorius*), lingual nerve (*nervus lingualis*), auricle-temporal nerve (*nervus auriculotemporalis*), mandibular-hyoid nerve (*nervus mylohyoideus*), and inferior alveolar nerve (*nervus alveolaris inferior*) [24, 25, 34]. In baboon and buff-bellied capuchins, this foramen was in line with the lateral lamina of the pterygoid process, in L’Hoest’s monkey, Angola colobus, and Celebes crested macaque slightly shifted medially in comparison to baboon and buff-bellied capuchins. In contrast, black-and-white ruffed lemur, ring-tailed lemur, and crowned lemur were present more medially to the lateral lamina of the pterygoid process if compare to the species mentioned above.

### Anaesthesia techniques

***Anaesthesia of the infraorbital nerve.*** The infraorbital nerve gives off the superior alveolar branches responsible for the innervation of the maxillary teeth and the terminal branches innervating the skin and mucous membrane of the nose, upper lip and lower eyelid.

**Fig. 4:**
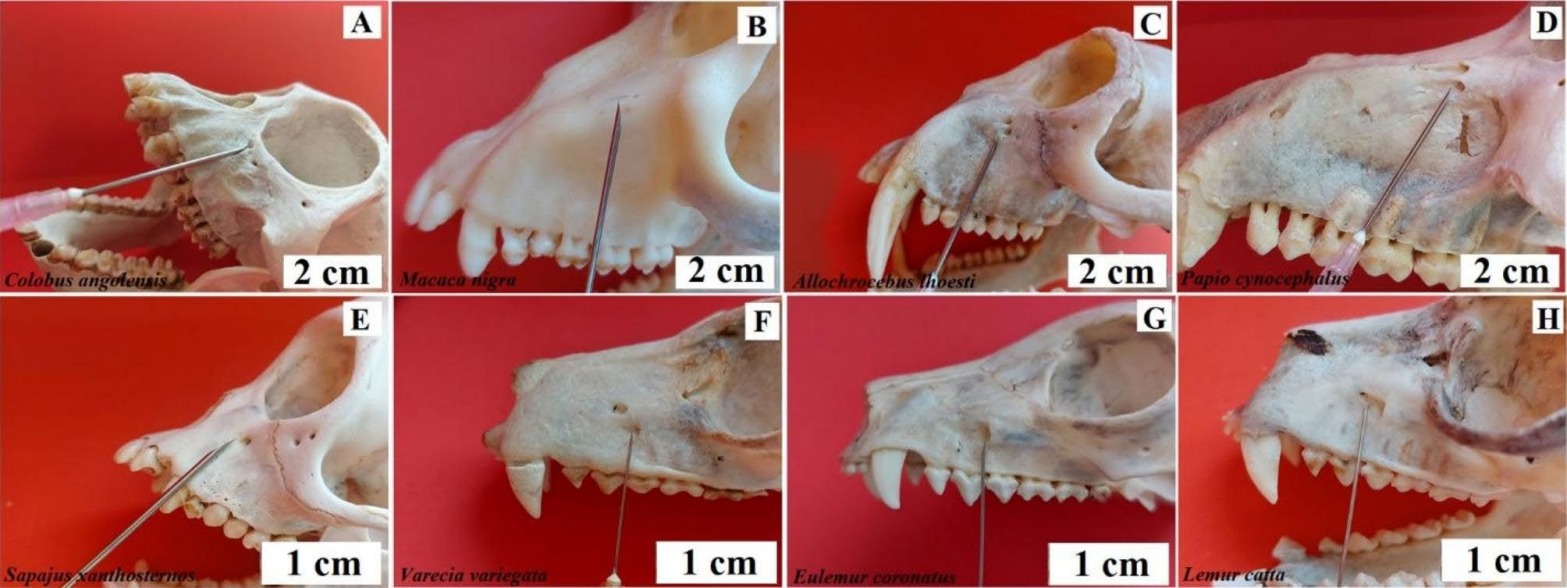
Anaesthesia of the infraorbital nerve. **A** – Angola colobus (*Colobus angolensis*), **B** – Celebes crested macaque (*Macaca nigra*), **C** – L’Hoest’s monkey (*Allochrocebus lhoesti*), **D** – Baboon (*Papio cynocephalus*), **E** – Buff-bellied capuchin (*Sapajus xanthostrenos*), **F** – Black-and-white ruffed lemur (*Varecia variegata*), **G** – Crowned lemur (*Eulemur coronatus*), **H** – Ring-tailed lemur (*Lemur catta*)

**Intraoral method.** The needle is inserted into the vestibule of the oral cavity, between the first and second incisor, and moved upwards and outwards, towards the pupil of the eye looking ahead [4]. Baboon - the needle insertion point is between the 3rd and 4th buccal teeth, the needle should be inserted at an angle towards the lower edge of the orbit (Fig. 4). Angola colobus - the needle insertion point is between the 1st and 2nd buccal teeth, pointing the needle vertically upwards (Fig. 4). Celebes crested macaque - the needle insertion point is at the level of the 4th buccal tooth, directing the needle vertically upwards (Fig. 4). L’Hoest’s monkey - the needle insertion site is between the 2nd and 3rd buccal teeth, directing the needle at an angle towards the lower edge of the orbit (Fig. 4). Buff-bellied capuchin - the needle insertion point is between the canine and the 1st buccal tooth, directing the needle at an angle towards the lower edge of the orbit (Fig. 4). Black-and-white ruffed lemur, ring-tailed lemur and crowned lemur - the needle insertion site is between the 2nd and 3rd buccal teeth pointing the needle straight up (Fig. 4).

**Fig. 5:**
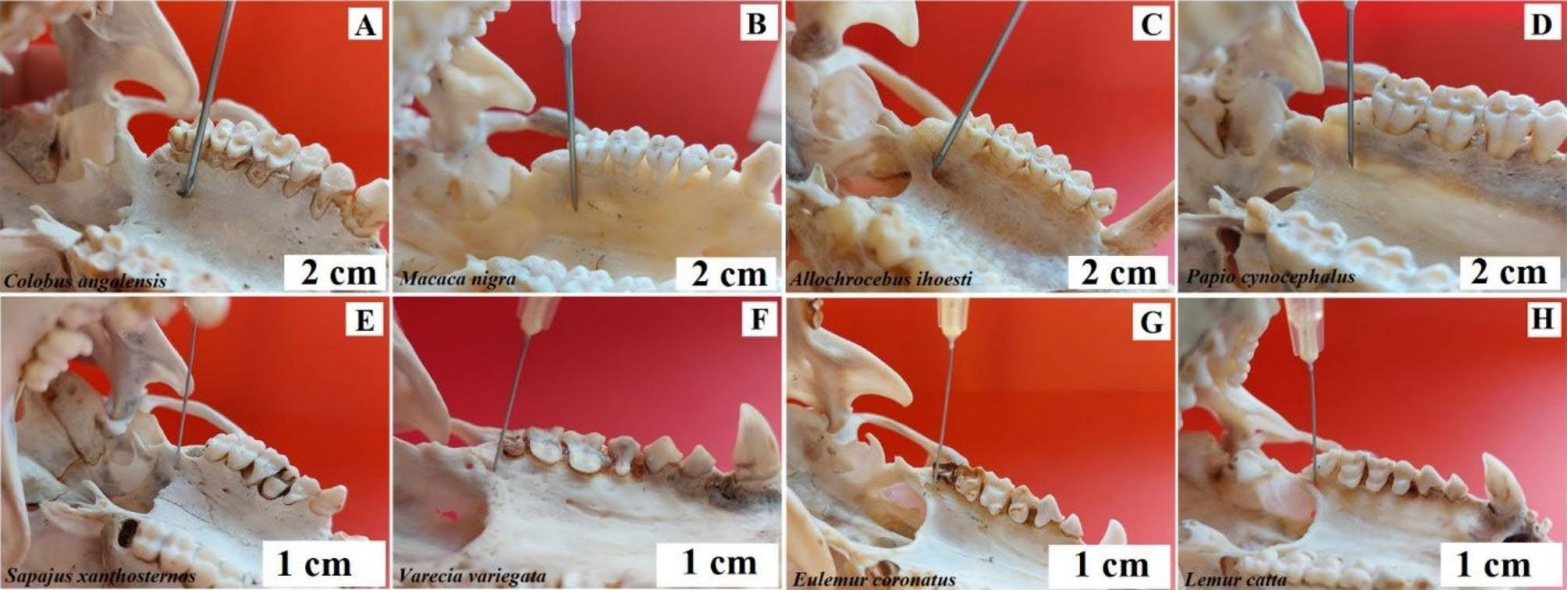
Anaesthesia of the greater palatine nerve. **A** – Angola colobus (*Colobus angolensis*), **B** – Celebes crested macaque (*Macaca nigra*), **C** – L’Hoest’s monkey (*Allochrocebus lhoesti*), **D** – Baboon (*Papio cynocephalus*), **E** – Buff-bellied capuchin (*Sapajus xanthostrenos*), **F** – Black-and-white ruffed lemur (*Varecia variegata*), **G** – Crowned lemur (*Eulemur coronatus*), **H** – Ring-tailed lemur (*Lemur catta*)

***Anaesthesia of the greater palatine nerve.*** The greater palatine nerve is responsible for innervating the mucous membrane of the hard palate. The needle is inserted at the level of the last molar [8]. It is directed diagonally backwards and upwards to the point of contact with the bone. Baboon, black-and-white ruffed lemur, buff-bellied capuchin, L’Hoest’s monkey, ring-tailed lemur, and Celebes crested macaque - the needle insertion site is behind the last buccal tooth (Fig. 5). Angola colobus – the needle insertion site is between the penultimate and last buccal teeth (Fig. 5). Crowned lemur - the needle insertion point is at the height of the last buccal tooth (Fig. 5).

**Fig. 6:**
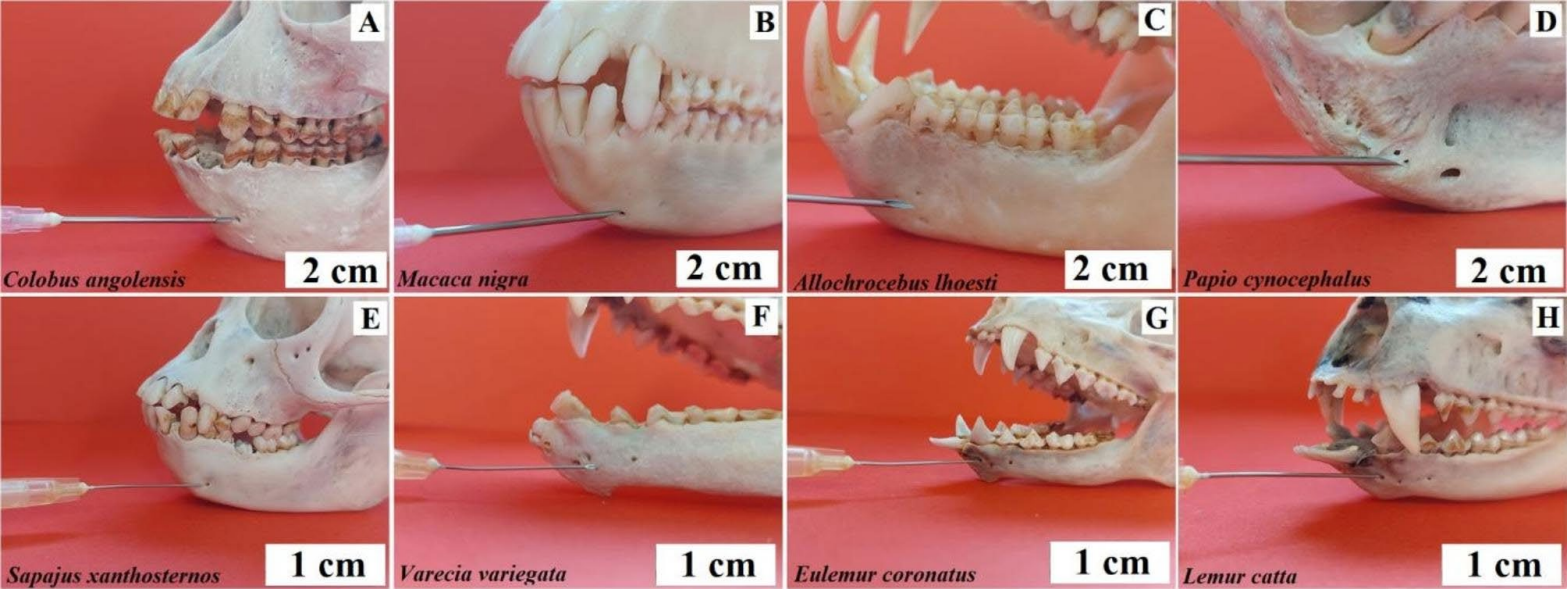
Anaesthesia of the mental nerve. **A** – Angola colobus (*Colobus angolensis*), **B** – Celebes crested macaque (*Macaca nigra*), **C** – L’Hoest’s monkey (*Allochrocebus lhoesti*), **D** – Baboon (*Papio cynocephalus*), **E** – Buff-bellied capuchin (*Sapajus xanthostrenos*), **F** – Black-and-white ruffed lemur (*Varecia variegata*), **G** – Crowned lemur (*Eulemur coronatus*), **H** – Ring-tailed lemur (*Lemur catta*)

***Anaesthesia of the mental nerve.*** The mental nerve is responsible for the sensory innervation of the skin of the chin and the mucous membrane of the lower lip. The needle is inserted through the skin after palpating the mental foramen with a finger. This method can be applied to all monkey species tested (Fig. 6).

**Fig. 7:**
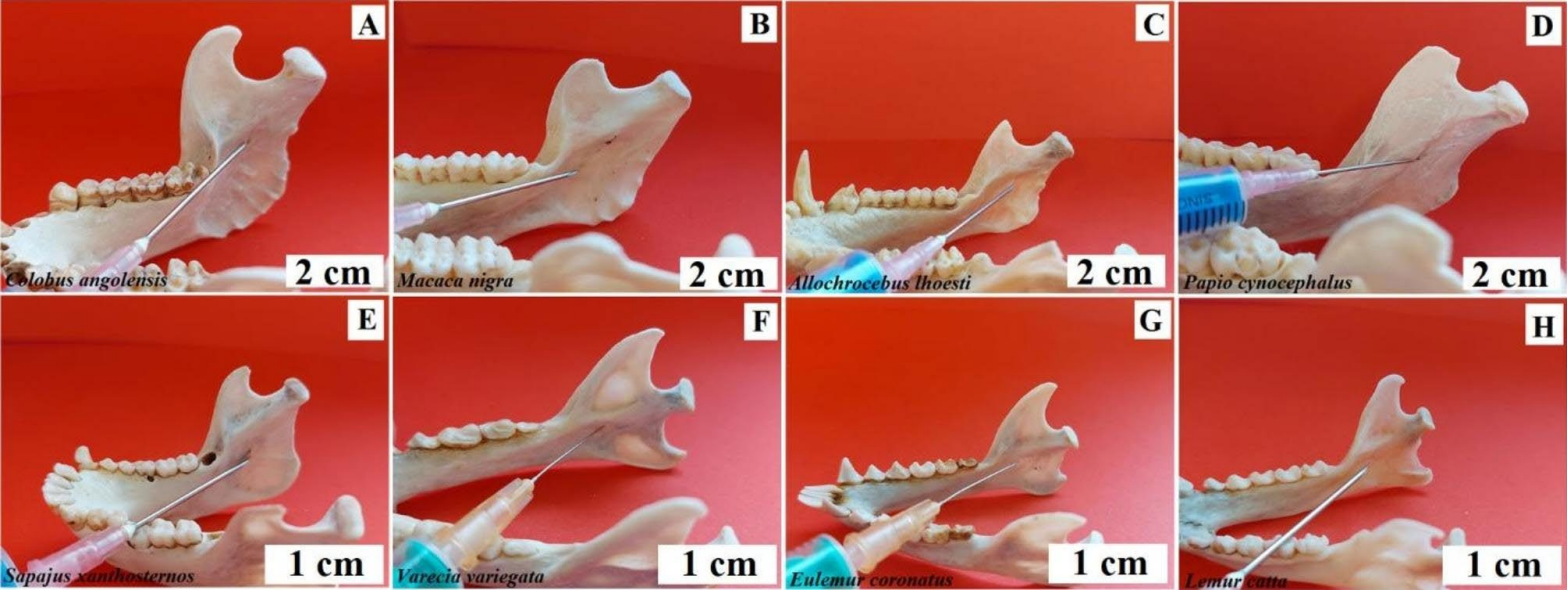
Anaesthesia of the alveolar inferior nerve. Intraoral method. **A** – Angola colobus (*Colobus angolensis*), **B** – Celebes crested macaque (*Macaca nigra*), **C** – L’Hoest’s monkey (*Allochrocebus lhoesti*), **D** – Baboon (*Papio cynocephalus*), **E** – Buff-bellied capuchin (*Sapajus xanthostrenos*), **F** – Black-and-white ruffed lemur (*Varecia variegata*), **G** – Crowned lemur (*Eulemur coronatus*), **H** – Ring-tailed lemur (*Lemur catta*)

***Anaesthesia of the inferior alveolar nerve.*** The inferior alveolar nerve is responsible for the sensory innervation of the teeth of the lower jaw. **Intraoral method**. The puncture point is on the inner surface of the mandible, 1 cm above the level of the masticatory surface of the lower molars. The needle is guided parallel to the occlusal plane of the lower teeth along the inner surface of the mandibular branch. Baboon, buff-bellied capuchin, L’Hoest’s monkey, and Angola colobus - the puncture site is at the level of the masticatory surface of the lower molars (Fig. 7). Black-and-white ruffed lemur, ring-tailed lemur, and Celebes crested macaque - the puncture site is 0.5 cm below the masticatory surface of the lower molars (Fig. 7). Crowned lemur - the puncture site is 0.3 cm below the masticatory surface of the lower molars (Fig. 7).

**Fig. 8:**
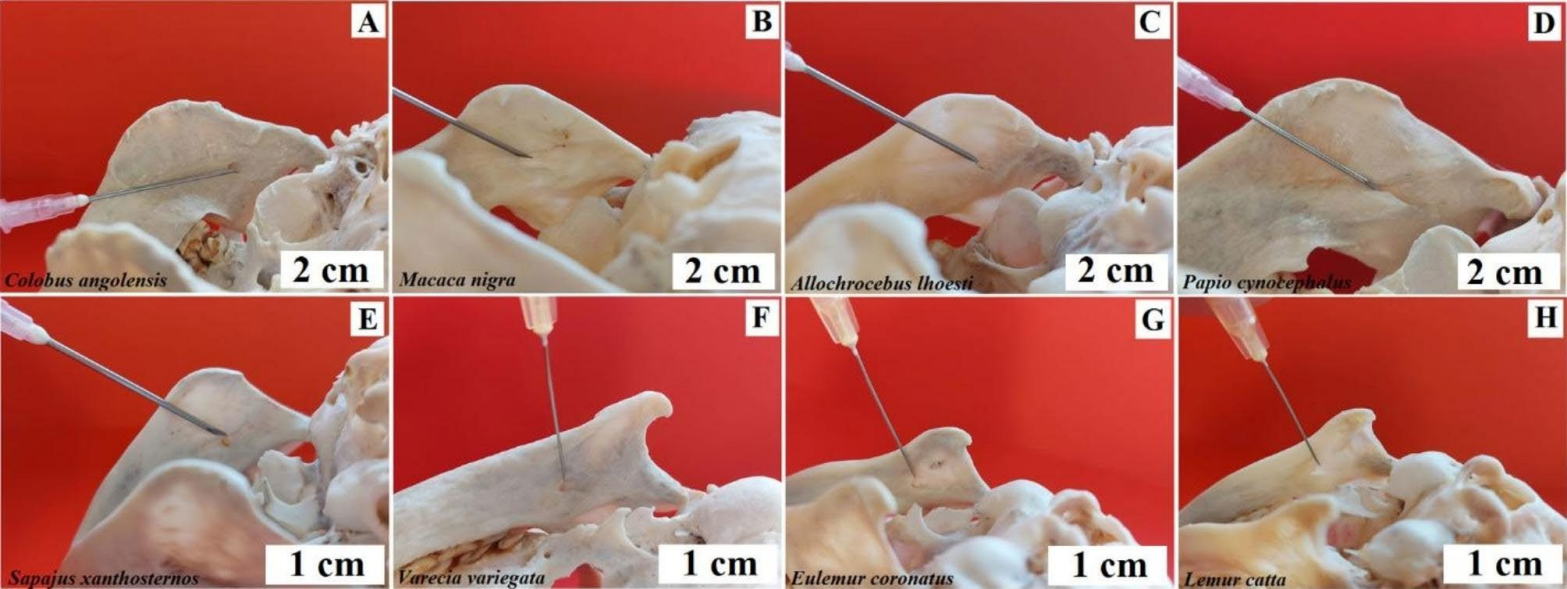
Anaesthesia of the alveolar inferior nerve. Extraoral method. **A** – Angola colobus (*Colobus angolensis*), **B** – Celebes crested macaque (*Macaca nigra*), **C** – L’Hoest’s monkey (*Allochrocebus lhoesti*), **D** – Baboon (*Papio cynocephalus*), **E** – Buff-bellied capuchin (*Sapajus xanthostrenos*), **F** – Black-and-white ruffed lemur (*Varecia variegata*), **G** – Crowned lemur (*Eulemur coronatus*), **H** – Ring-tailed lemur (*Lemur catta*)

**Extraoral method.** Bottom access. The needle is inserted under the lower edge of the mandible 2 cm anteriorly from its angle, leading it along the inner surface of the edge of the mandible, parallel to its posterior edge. Baboon, L’Hoest’s monkey, and Angola colobus - needle insertion site from the notch in front of the angle of the mandible directing the needle caudally (Fig. 8). Celebes crested macaque and buff-bellied capuchin - puncture site from the notch in front of the mandibular angle, directing the needle vertically or slightly backwards. Black-and-white ruffed lemur, ring-tailed lemur, and crowned lemur - puncture site from the notch in front of the mandibular angle vertically upwards (Fig. 8).

**Fig. 9:**
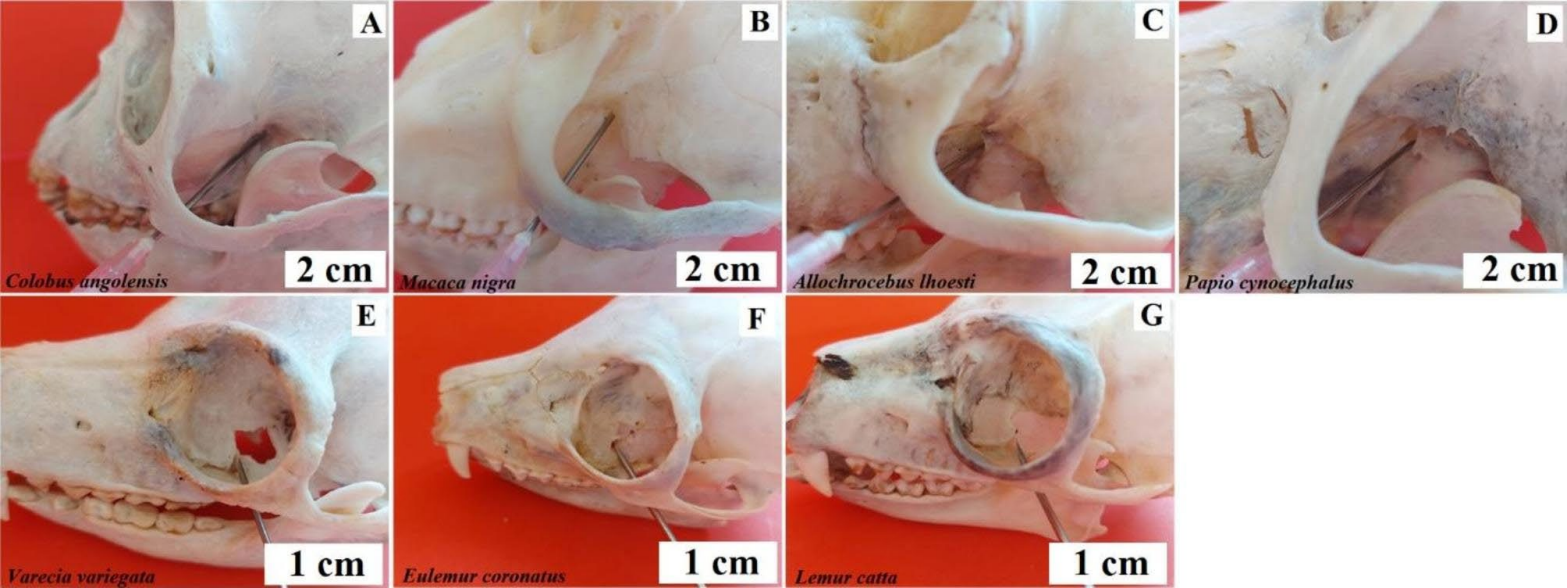
Anaesthesia of the maxillary nerve. The method is under the zygomatic arch. **A** – Angola colobus (*Colobus angolensis*), **B** – Celebes crested macaque (*Macaca nigra*), **C** – L’Hoest’s monkey (*Allochrocebus lhoesti*), **D** – Baboon (*Papio cynocephalus*), **E** – Black-and-white ruffed lemur (*Varecia variegata*), **F** – Crowned lemur (*Eulemur coronatus*), **G** – Ring-tailed lemur (*Lemur catta*)

**Anaesthesia of the maxillary nerve.** The maxillary nerve emerges from the round foramen and gives off the pterygopalatine nerves and the zygomatic nerve. Then it passes through the inferior orbital fissure into the orbit, and from there, it passes into the infraorbital canal as the infraorbital nerve. The zygomatic nerve is responsible for innervating the skin of the lateral forehead, the front of the temple, and the cheek in the zygomatic region. The pterygopalatine nerves reach the pterygopalatine ganglion and form the sensory root. **The method under the zygomatic arch.** The puncture needle should be directed at the intersection of the line extending the posterior edge of the frontal process of the zygomatic bone with its lower edge. The needle is inserted with the mouth closed through the buccal muscle to a depth of 2 to 3 cm until contact with the bone is achieved. It is then moved upwards and slightly posteriorly along the posterior surface of the maxillary tubercle. At a depth of 5 cm, the tip of the needle reaches the anterior surface of the greater wing of the sphenoid at the round foramen. Baboon, Angola colobus, L’Hoest’s monkey, and Celebes crested macaque - the method given above may be used (Fig. 9). And in buff-bellied capuchins, this method cannot be used due to the structure of the skull. The black-and-white ruffed lemur, ring-tailed lemur, and crowned lemur cannot be adapted from human medicine due to the closer similarity of this part of their skull to that of a dog. Looking at the method of anaesthetizing this nerve in dogs, the needle should be inserted in the angle formed by the zygomatic arch and the caudal part of the maxilla, directing the needle perpendicularly to the pterygopalatine fossa [35] (Fig. 9).

**Fig. 10:**
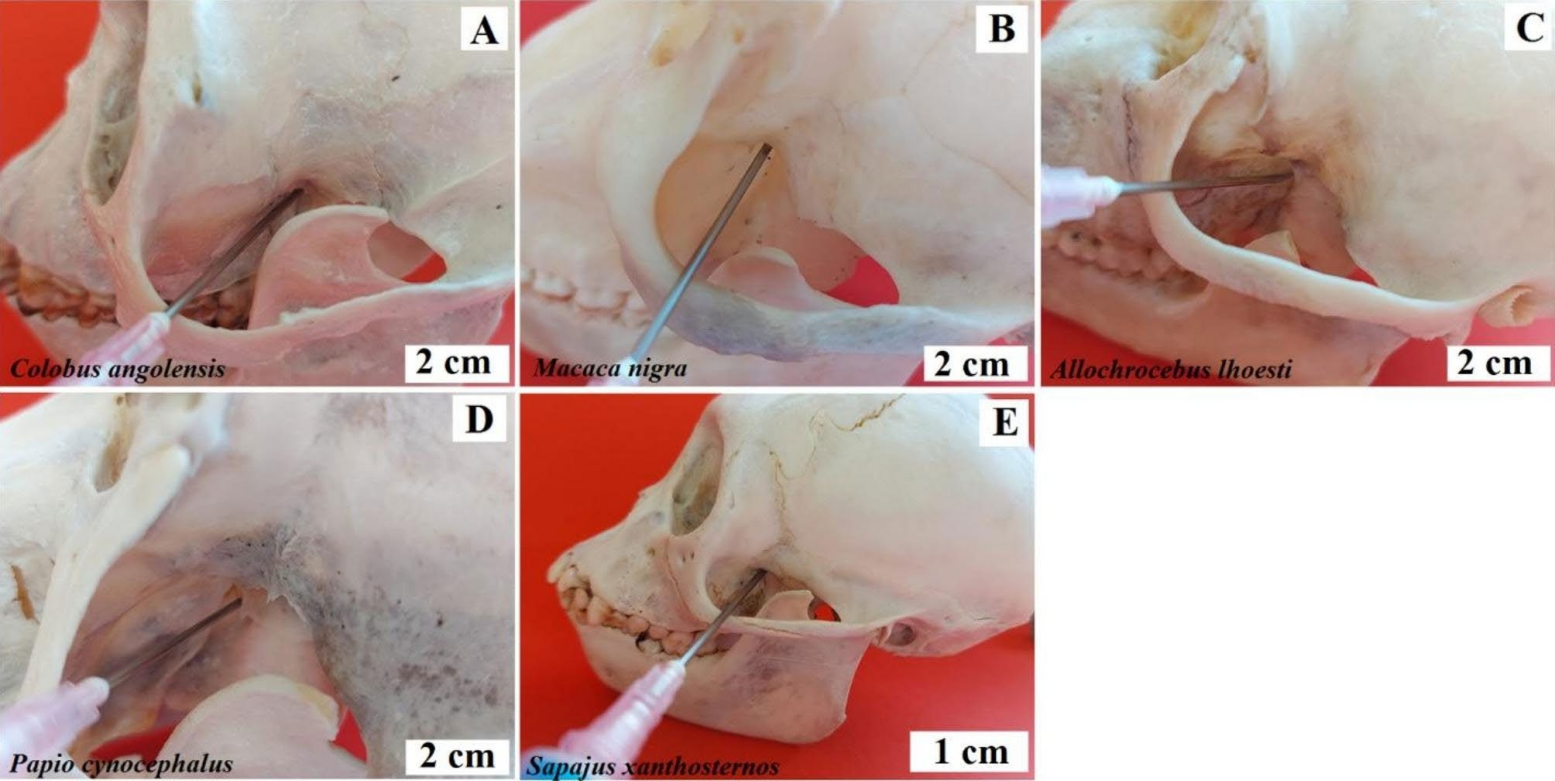
Anaesthesia of the maxillary nerve. The method over the zygomatic arch. **A** – Angola colobus (*Colobus angolensis*), **B** – Celebes crested macaque (*Macaca nigra*), **C** – L’Hoest’s monkey (*Allochrocebus lhoesti*), **D** – Baboon (*Papio cynocephalus*), **E** – Buff-bellied capuchin (*Sapajus xanthostrenos*)

**The method over the zygomatic arch.** The puncture point lies at the angle between the zygomatic bone and the posterior edge of the frontal process. The puncture direction is initially from the top and side towards the centre and down, and after reaching the maxillary tuber slightly up and back. Baboon and Celebes crested macaque - the needle should be directed caudally-medially in the horizontal plane. Angola colobus - the needle should be directed caudal-medial in the horizontal plane, slightly downwards (Fig. 10). L’Hoest’s monkey and buff-bellied capuchin - the needle insertion site is slightly caudal from the designated point, directing the needle medially and further as in the species described above (Fig. 10). Black-and-white ruffed lemur, ring-tailed lemur, and crowned lemur - this technique cannot be used due to the structure of the temporal fossa.

**Fig. 11:**
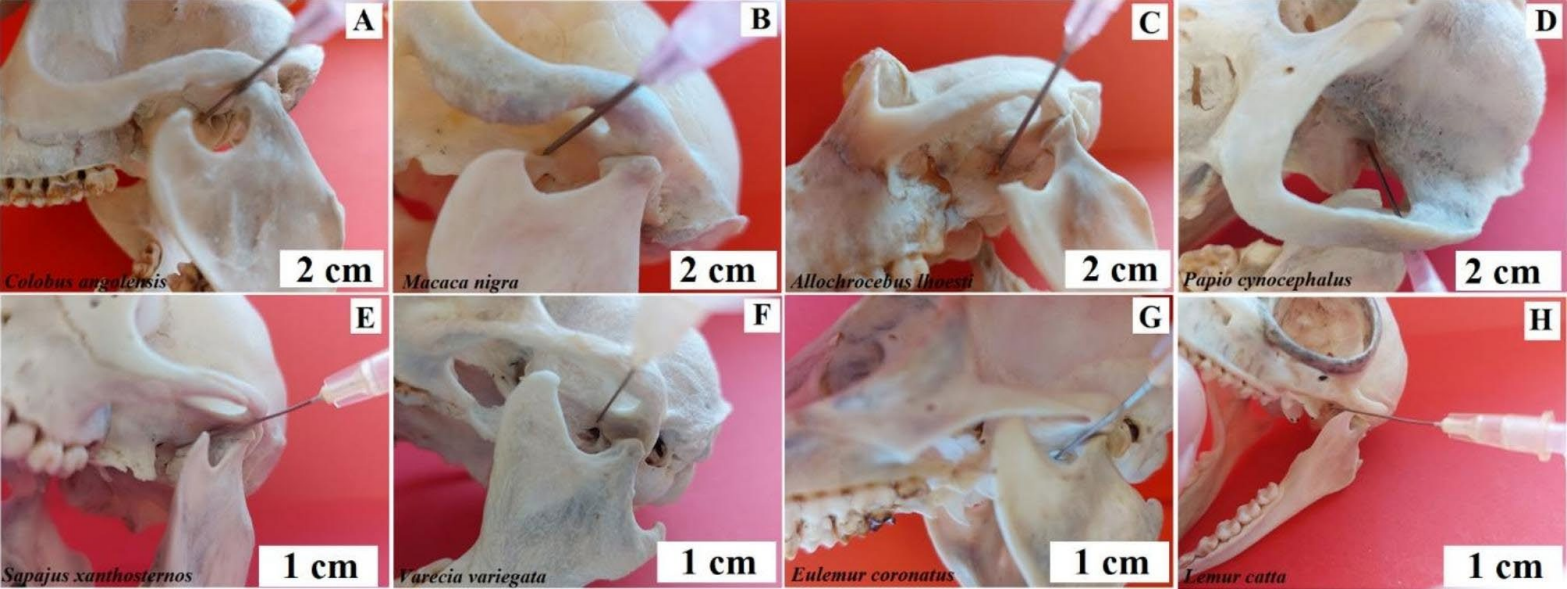
Anaesthesia of the maxillary nerve at the oval foramen. The method is under the zygomatic arch. **A** – Angola colobus (*Colobus angolensis*), **B** – Celebes crested macaque (*Macaca nigra*), **C** – L’Hoest’s monkey (*Allochrocebus lhoesti*), **D** – Baboon (*Papio cynocephalus*), **E** – Buff-bellied capuchin (*Sapajus xanthostrenos*), **F** – Black-and-white ruffed lemur (*Varecia variegata*), **G** – Crowned lemur (*Eulemur coronatus*), **H** – Ring-tailed lemur (*Lemur catta*)

***Anaesthesia of the mandibular nerve at the oval foramen.*** The branches of the mandibular nerve supply sensory fibres around the temples, cheeks and the area of ​​the mouth from the side of the mandible. It is also responsible for the movements of the masticatory muscles. **The method under the zygomatic arch.** The puncture site is 1-1.5 cm in front of the jaw joint. The needle is inserted with the jaws slightly open and above the mandibular notch, strictly in the frontal plane to the base of the pterygoid process of this sphenoid bone. Baboon, Angola colobus, Celebes crested macaque, L’Hoest’s monkey, and buff-bellied capuchin - the needle should be pointed horizontally, medially (Fig. 11). Black-and-white ruffed lemur, ring-tailed lemur, and crowned lemur - the needle should be pointed horizontally, medially and slightly posteriorly (Fig. 11).

**Fig. 12:**
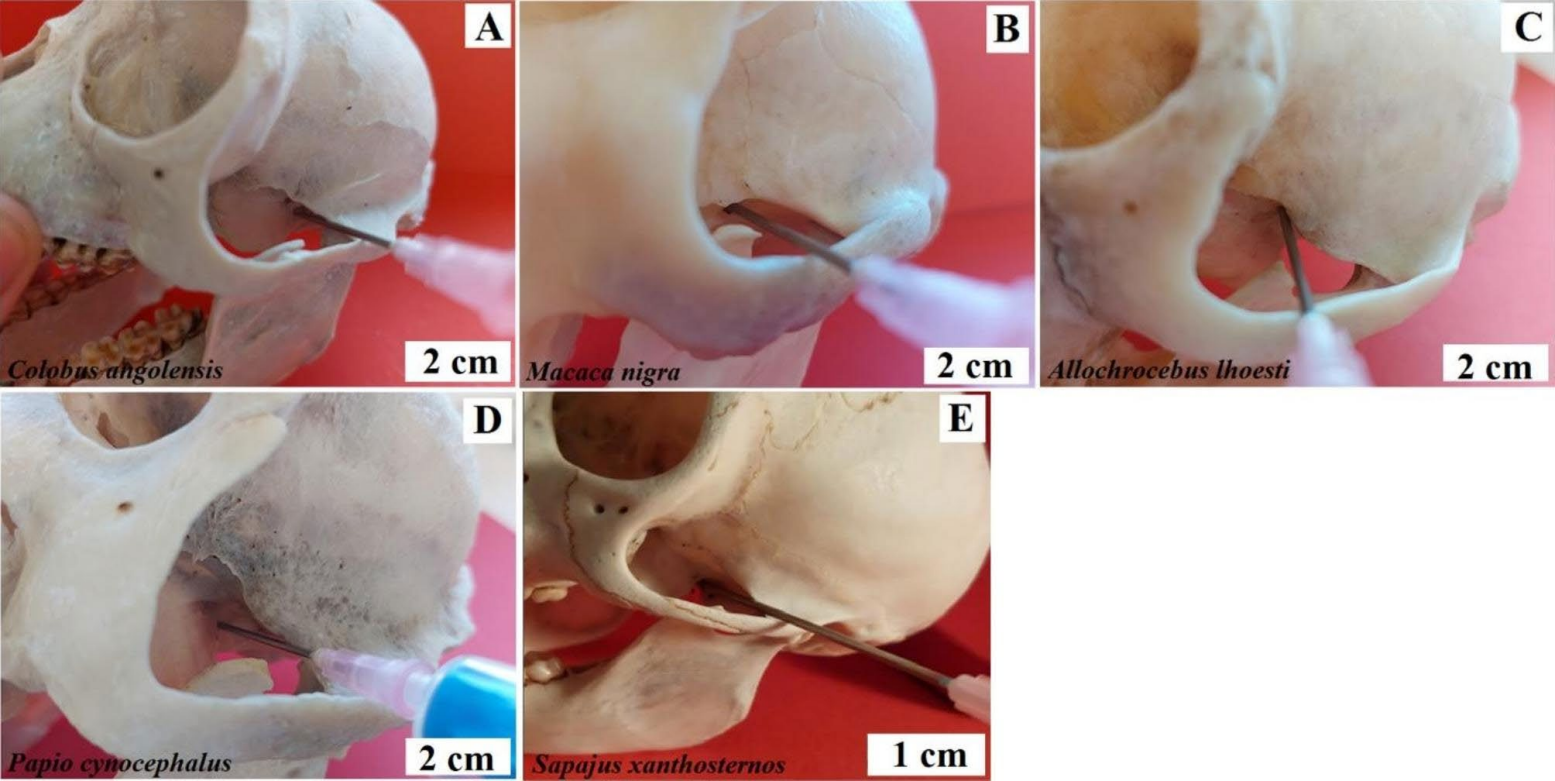
Anaesthesia of the mandibular nerve at the oval foramen. The method over the zygomatic arch. **A** – Angola colobus (*Colobus angolensis*), b – Celebes crested macaque (*Macaca nigra*), **C** – L’Hoest’s monkey (*Allochrocebus lhoesti*), **D** – Baboon (*Papio cynocephalus*), **E** – Buff-bellied capuchin (*Sapajus xanthostrenos*)

**The method over the zygomatic arch.** The puncture site is above the highest point of the arch. Initially, the needle is inserted in the frontal plane. After reaching the base of the pterygoid process of the sphenoid bone, the needle is directed slightly posteriorly and further 0.5 cm deeper. Baboon, Angola colobus, Celebes crested macaque, and L’Hoest’s monkey. The technique described above was used (Fig. 12). Buff-bellied capuchin - the needle should be pointing slightly downwards (Fig. 12). Black-and-white ruffed lemur, ring-tailed lemur, and crowned lemur - this technique cannot be used due to the structure of the temporal fossa.

The presented results show that perineural injections for block anaesthesia of the relevant structures are possible in terms of the skull anatomy of selected primates. This makes it possible to reduce postoperative pain, limit the dose of general anaesthetics and reduce the number of potential complications associated with the use of systemic anaesthetics [36]. This is especially important in patients who, for several reasons, have an increased risk of complications or a lower quality of life. The use of anaesthesia in the area of the oral cavity in postoperative pain therapy may have a beneficial effect on the appetite of animals that usually have problems with food intake due to pain [37].

The size of the cranial foramina is a reference to the approximate size of the nerve exiting it [28]. This is important because the dose of the anaesthetic substance should be adjusted to the area of anaesthesia (i.e. the larger the nerve, the higher the dose should be because the branches of a given nerve cover a larger area of soft tissues - the principle works both ways). While the dose of anaesthesia can be standardised, side effects and possible complications may be related to the dose of the anaesthetic used. Therefore, it is crucial to consider the use of a smaller volume of the drug in the case of perineural analgesia of smaller nerves, while achieving anaesthesia of the given area. It should also be remembered that administering too little anaesthesia may limit the area of anaesthesia or even not lead to it [9].

The presented methods are similar to those used in humans or animals. In small animals, anaesthesia of the *n. maxillaris, n. alveolaris inferior* and *n. infraorbitalis* is most commonly performed [13–15]. However, anatomical differences in the skulls of primates prevent some injections from being performed identically. Due to the structure of the temporal bone and the location of the maxillary foramen, it was impossible to insert the needle using the method under the zygomatic arch to anaesthetize the maxillary nerve in the crested macaque and the method over the zygomatic arch in lemurs. A similar problem occurred in this superfamily in the insertion of the needle near the oval foramen by the method over the zygomatic arch to anaesthetize the mandibular nerve. In addition, many differences related to injection in the oral cavity have been demonstrated. The guidance of the needle in the case of methods of anaesthesia of the infraorbital, mental and greater palatal nerves differed in terms of the puncture site about the buccal teeth. In addition, the difference in the number of infraorbital and mental foramen among species and individuals of the same species poses a problem in the case of anaesthesia of a given region of the head, and it is recommended to anaesthetize the proximal nerve to them, i.e. the maxillary nerve and the inferior alveolar nerve [7, 38].

## Conclusion

The study aimed to check whether there are large discrepancies in the topography of cranial foramina among the examined individuals belonging to the order of primates, as well as to check whether perineural anaesthesia techniques performed in human medicine can be used in these species. The study showed that there are significant species differences between the tested individuals despite their close relationship. It was also found possible to use anaesthesia techniques performed in humans with some modifications resulting from the specific structure of the skull of individual species. The above work shows that although the presented species belong to exotic animals and the knowledge about them is not as extensive as about companion or farm animals, it is possible to perform perineural injections in them and it can be used to provide them with pain comfort by veterinarians working in zoos and national parks. However, the angles of the needle placement are likely to vary with an individual (and within an individual based on age). Thus our proposed routes for and placement of needles should be interpreted as guidance and less as a directive.

## Data Availability

The authors confirm that our article type does not require a data availability statement.
